# An International Survey on the Use of a Polyrevitalizing Solution With or Without Other Aesthetic Procedures in the Daily Practice of Aesthetic Physicians

**DOI:** 10.1111/jocd.16623

**Published:** 2025-01-22

**Authors:** Ferial Fanian, Gabriela Casabona, Emanuele Bartoletti, Hugues Cartier, Marina Landau, Hassan Galadari, Fotini Bageorgou, Alexandra Ogilvie, Elina Theodorakopoulou, Ariana Arteaga, Hilal Gokalp, Philippe Hamida Pisal, Ingrid Gerhke Lopez, Gabriel Rucinski, Myriam Chebbah, Solenn Le Clanche, Alice‐Anaïs Varlet, Natalia Sukmanskaya, Hanane Issa, Valerie Philippon, Alexander Stratigos

**Affiliations:** ^1^ Scientific Department Laboratoires FILLMED Paris France; ^2^ Ocean Clinic Marbella Malaga Spain; ^3^ Department of Aesthetic Medicine Fatebenefratelli Isola Tiberina‐Gemelli Rome Italy; ^4^ Centre Médical Saint Jean Arras France; ^5^ Arena Dermatology and Department of Plastic Surgery Shamir Medical Center Be'er Ya'akov Israel; ^6^ College of Medicine and Health Sciences at United Arab Emirates University Al Ain UAE; ^7^ For Better Skin Clinic Athens Greece; ^8^ Peeling Department Andreas Sygros Hospital of Dermal & Venereal Disease Athens Greece; ^9^ SkinConcept Munich Germany; ^10^ Pretty You Dermatology Clinic Athens Greece; ^11^ Clinic of Dr Ariania Arteaga Valencia Spain; ^12^ HG Clinic Istanbul Turkey; ^13^ PHP Clinic London UK; ^14^ Hout Klinik Dermatologica Mexico City Mexico; ^15^ BELLAMED Clinic Elblag Warmia‐Masuria Poland; ^16^ Medical Affairs Department Public Health Expertise Paris France; ^17^ Department of Dermatology University of Athens School of Medicine, Andreas Sygros Hospital Athens Greece

**Keywords:** combination therapy, NCTF135HA, Polyrevitalization

## Abstract

**Objective:**

NCTF135HA, a versatile polyrevitalizing solution, is a potent agent for enhancing skin quality, radiance, moisture, vitality, and diminishing fine wrinkles caused by aging factors. Data demonstrate a divergence in its application from skin quality enhancement to treatment of vitiligo lesions. To know more precisely about the protocol of use among providers, alone or in combination with other procedures, we performed an international survey.

**Method:**

A Steering Committee of dermatologists, aesthetic surgeons, and physicians developed a 32‐question questionnaire based on a literature review. Hosted online from January to March 2024, it targeted healthcare professionals experienced in polyrevitalization. Responses were analyzed anonymously and reported descriptively.

**Results:**

Practitioners adopt a balanced approach: half of their patients receiving classic Polyrevitalization (NCTF135HA alone) and the other half undergoing combination therapy (NCTF135HA with another aesthetic procedure). Most administer NCTF135HA across multiple sessions, typically three (39.7%). In combination therapy, 55.5% of practitioners use NCTF135HA for medical purposes, targeting Melasma (53.1%) and Rosacea (45.0%) for instance. Aesthetic use is prevalent, with 94.2% employing NCTF135HA for skin wrinkles (79.9%), and refreshment, rejuvenation, and hydration (73.4%). Combining NCTF135HA with hyaluronic acid (71.2%) and botulinum toxin (49.1%) is common, alongside microneedling (50.2%), peelings (32.4%), and fractional radiofrequency (25.6%).

**Limitations and Conclusion:**

Our survey showed a homogenous distribution of NCTF135HA utilization, indicating a consensus on its application across diverse demographics. This consistency highlights its widespread acknowledgment and efficacy in various aesthetic and clinical settings. Limitations include a large respondent group from Mexico and many respondents with less than 10 years of experience.

## 1 Introduction

Polyrevitalization, previously referred as “mesotherapy” or “Mesolift,” is a minimally invasive procedure that aims to improve skin quality, rejuvenate and revitalize the skin by injecting a cocktail of numerous nutritive compounds into the dermis [1]. As aged skin requires a supply of diverse substrates essential for optimal cellular function, polyrevitalization agents have evolved towards sophisticated polycomponent preparations to fulfill this need [2]. At the cellular level, polyrevitalization agents create an optimal physiological environment for enhancing the biosynthetic capabilities of fibroblasts. This, in turn, promotes cellular interaction and boosts the production of collagen, elastin, and glycosaminoglycans such as hyaluronic acid (HA) [3, 4]. Polyrevitalization agents may include HA, vitamins, minerals, amino acids, cytokines, growth factors, co‐enzymes, nucleic acids, and antioxidants [4, 5].

Polyrevitalization procedure is increasingly preferred for its efficiency and safety compared to the invasive options [5, 6, 7]. Indeed, polyrevitalization is an adaptable treatment option which can enhance skin quality with minimal discomfort and side effects [8]. In recent years, its use in combination with various other treatments has increased. Numerous combinations featuring polyrevitalizing products have been documented in the literature, showcasing their efficacy across different indications. Notably, the combination of HA fillers and botulinum toxin stands out as one of the most practiced methods, offering a comprehensive solution for addressing various layers of facial tissues and delivering satisfactory outcomes for patients navigating the signs of aging [6, 8, 9, 10, 11, 12]. Other notable combinations include HA fillers paired with laser‐radiofrequency‐intense pulsed light and mesotherapy for facial rejuvenation [9], as well as deep fractional radiofrequency (FR) alongside bipolar FR and mesotherapy for tackling skin laxity [13, 14]. Autologous platelet concentrates (APCs) combined with microneedling mesotherapy techniques have been lauded for their effectiveness in rejuvenating the face [15], while growth factors in conjunction with mesotherapy microneedling have shown promise in achieving similar results [16]. Platelet‐rich plasma (PRP) has been combined with mesotherapy to target conditions such as melasma [17] and alopecia [18, 19], while UV light paired with LED light, chemical peels, stem cells, retinoids, and mesotherapy microneedling has been explored for addressing stretch marks, cellulite, alopecia, and vitiligo [20]. Additionally, PRP in combination with plastic surgery and mesotherapy has been utilized for wound healing purposes [19]. These combinations exemplify the versatility and potential of polyrevitalization in enhancing aesthetic and dermatologic outcomes across a spectrum of indications.

Among the indications for which polyrevitalization combination is used, skin rejuvenation, wrinkles, and photodamaged skin treatment are the most common [5, 9, 15, 21, 22, 23, 24]. NCTF135HA is a state‐of‐the‐art polyrevitalizing product, featuring a unique blend of 60 nutritive components, including vitamins, minerals, amino acids, nucleotides, coenzymes, antioxidants, and HA [25]. When used alone, this product has proven its efficacy as a skin booster to temporarily improve its quality, brightness, hydration, fatigue [26], and fine wrinkles associated with endogenous and environmental aging [25], both in vitro and *in vivo* [25, 26, 27, 28, 29]. Moreover, in line with the trend of combined therapy, NCTF135HA remains consistent with this approach and is frequently employed in combination with other treatments mentioned earlier to maximize its benefits [8, 25, 29, 30]. Although NCTF135HA is used in combination in practice, there is a lack of consensus regarding injection protocols, session frequency, intervals between sessions and indications for NCFT135HA in combination worldwide.

Thus, the objective of the present study was to investigate the NCTF135HA real‐life practice when used in combination therapy among the international experts in skin quality treatment.

## 2 Materials and Methods

A questionnaire was drafted by a Steering Committee (SC) composed of dermatologists, plastic surgeons, and aesthetic doctors from 10 different countries. To identify the questions to be addressed, a review of scientific literature was conducted using PubMed/Medline, Cochrane, Lissa, and CISMeF databases, as well as gray literature (learned societies, patient associations, etc.). This literature review allowed to accurately define the borders of the topics to be included in the survey and to be aligned with the routine clinical practices and the current perception of the NCTF135HA reallife practice around the world.

The questionnaire was hosted online on a dedicated secure platform open from January 2024 to March 2024, and included 32 main questions divided into 2 thematic sections: (1) NCTF135HA in practice alone and (2) NCTF135HA utilization in combination. Of the 32 questions, 7 were open‐field responses, 20 were multiple‐choice, and 5 were single‐choice questions.

The responding healthcare professionals had to be practitioners performing polyrevitalization procedures. The link to access the questionnaire was distributed by the SC members to their professional networks and through local distributors among the practitioners who use NCTF135HA in their daily practice. All the responses were analyzed on Excel software (calculation and figures) in March 2024, in aggregate only, while respecting the anonymity of the respondents. The results are descriptively reported.

## 3 Results

### 3.1 Description of the Respondents

A total of 507 practitioners participated in the international survey, with the majority identified as aesthetic doctors (52.1%, 264/507) and dermatologists (18.1%, 92/507) (Table 1). Most respondents (68.8%, 349/507) reported having less than 10 years of experience, while 7.3% (37/507) indicated over 20 years of practice (Table 1). The survey drew participation from practitioners in 60 different countries, primarily Europe (50.3%) and the Americas (43.6%) (Table 1). On average, practitioners admit 45% of the patients aged between 35 and 55 years old and 30% between 25 and 35 years old (Table 1). 54.3% (222/409) of the respondents reported receiving 10 to 30 patients per week. 74% of their activity has been showed on aesthetic procedures rather than the pure medical treatments (Table 1). Furthermore, among ten patients treated, respondents typically treated 80% females and 21% males (Table 1).

**TABLE 1:** Characteristics of the respondents and their patients.

Practitioner	TOTAL *n* = 507	%
Specialty (single choice question)	*N* = 507	%
Aesthetic doctor	264	52.1
Dermatologist	92	18.1
Surgeon	49	9.7
Dentist	46	9.1
General practitioner	22	4.3
Aesthetic technician	11	2.2
Aesthetic nurse	9	1.8
Other	14	2.8
Gynecologist	3	0.6
Alternative practitioner	3	0.6
ENT (Ear, Nose, and Throat)	2	0.4
Rehabilitation	1	0.2
Traditional Chinese Medicine	1	0.2
Orthopedic	1	0.2
Pharmacist	1	0.2
Pathologist	1	0.2
Internal medicine	1	0.2
Years of experience (mean [min‐max]) (open‐field question)	9 [1–44]	
< 10 years	349	68.8
10–20 years	121	23.9
> 20 years	37	7.3
Continents (single choice question)	*N* = 507	%
Europe	255	50.3
Americas	221	43.6
Asia and Oceania	21	4.1
Africa	10	2.0
**Patients**		
Age* (open‐field response) (*N* = 352)	%	
< 18 years	9	
18–25 years old	19	
25–35 years old	30	
35–55 years old	45	
55–65 years old	29	
> 65 years old	16	
Patients visited/week (single choice question)	*N* = 409	%
≤ 10	11	2.7
10–30	222	54.3
30–50	97	23.7
> 50	79	19.3
Percentage of aesthetic procedures* (%) (open‐field question)	*N* = 368	%
Mean	7.4	
< 50% of total patients	67	18.2
50%–70% of total patients	78	21.2
> 70% of total patients	223	60.6
Gender on aesthetic procedures* (open‐field question) (*N* = 375)	%	
Female	80	
Male	21	
Binary	5	

* Respondents provided their answers based on data from an average of 10 patients for each category.

### 3.2 Physicians' Attitude Towards the Use of NCTF135HA in Practice

**FIGURE 1:**
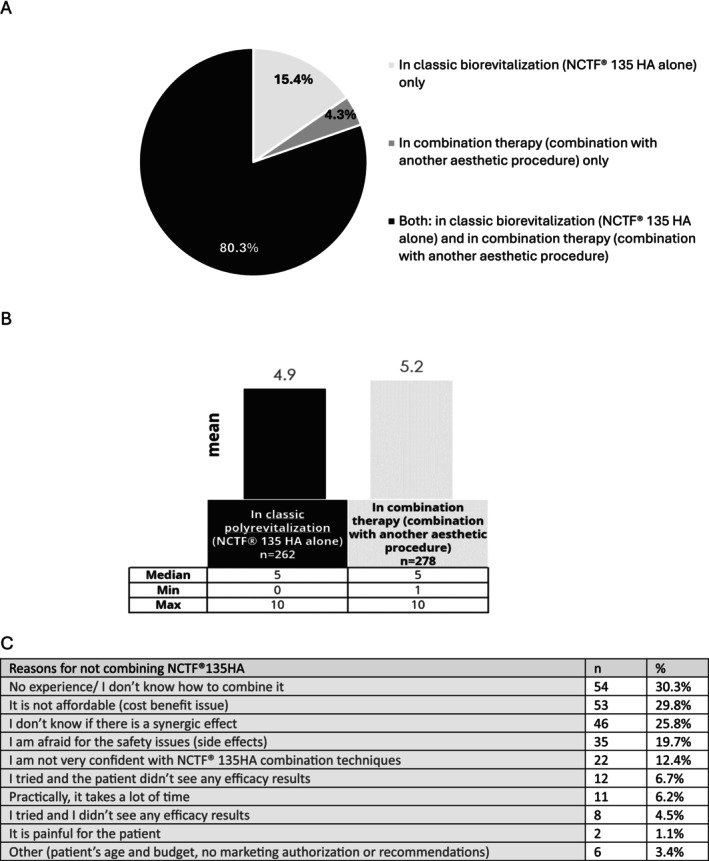
NCTF135HA use in practice. (A) Distribution of the use in classic polyrevitalization and/or in combination therapy (*n* = 371; single choice question); (B) Number of patients put on classical polyrevitalization and on combination therapy among 10 interventions (*n* = 278; open‐field response); (C) Reasons for not combining NCTF135HA (*n* = 178; open‐field response).

Among the practitioners surveyed, 80.3% (298/371) reported using NCTF135HA in both classic polyrevitalization and combination therapy, while 15.4% (57/371) exclusively employed NCTF135HA in classic polyrevitalization alone (Figure 1A). Practitioners using NCTF135HA in both applications reported dividing their patients evenly between classic polyrevitalization and combination therapy (Figure 1B). Reasons for not combining NCTF135HA included lack of experience (30.3%, 54/178), cost concerns (29.8%, 53/178), and uncertainty about synergistic effects (25.8%, 46/178) (Figure 1C).

### 3.3 NCTF135HA Used in Combination

Among respondents, 46.5% (132/284) became aware of NCTF135HA's combination with other procedures through congresses, while 41.5% (118/284) gained knowledge through their own professional experiences. Additionally, 41.2% (117/284) learned of this practice through trainings (Table 2).

**TABLE 2:** How respondents became aware of NCTF135HA combination practice (multiple‐choice question).

	*n*	%
From a congress	132	46.5
From your own professional experience	118	41.5
From trainings	117	41.2
From literature/publications	99	34.9
From a colleague	86	30.3
From master classes	62	21.8
From social media	39	13.7
From a patient	5	1.8
Other (company support and representatives, presentations, and events)	21	7.4

#### 3.3.1 NCTF 135HA Used in Combination for Medical Purposes

**FIGURE 2:**
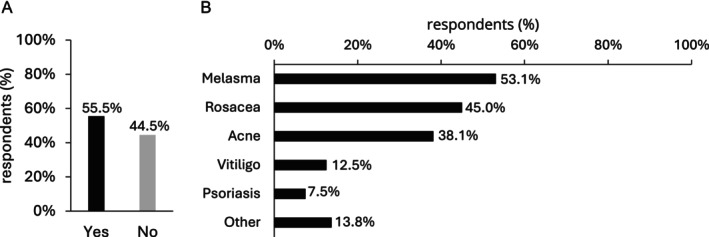
NCTF135HA in combination for medical purposes. (A) Percentage of respondents using NCTF135HA in combination for medical purposes (*n* = 292; single‐choice question); (B) Indications for the use of NCTF135HA in combination for medical purposes (*n* = 160; multiple‐choice question), Other: Alopecia, skin treatment, skin aging, scars treatment, and dermatologic treatment.

Of the respondents using NCTF135HA in combination therapy, 55.5% (162/292) stated they were employing it for medical purposes (Figure 2A). Among these, the primary conditions being addressed were melasma (53.1%, 85/160), rosacea (45.0%, 72/160), and acne (38.1%, 61/160) (Figure 2B).

#### 3.3.2 NCTF135HA Used in Combination for Aesthetic Purposes

**FIGURE 3:**
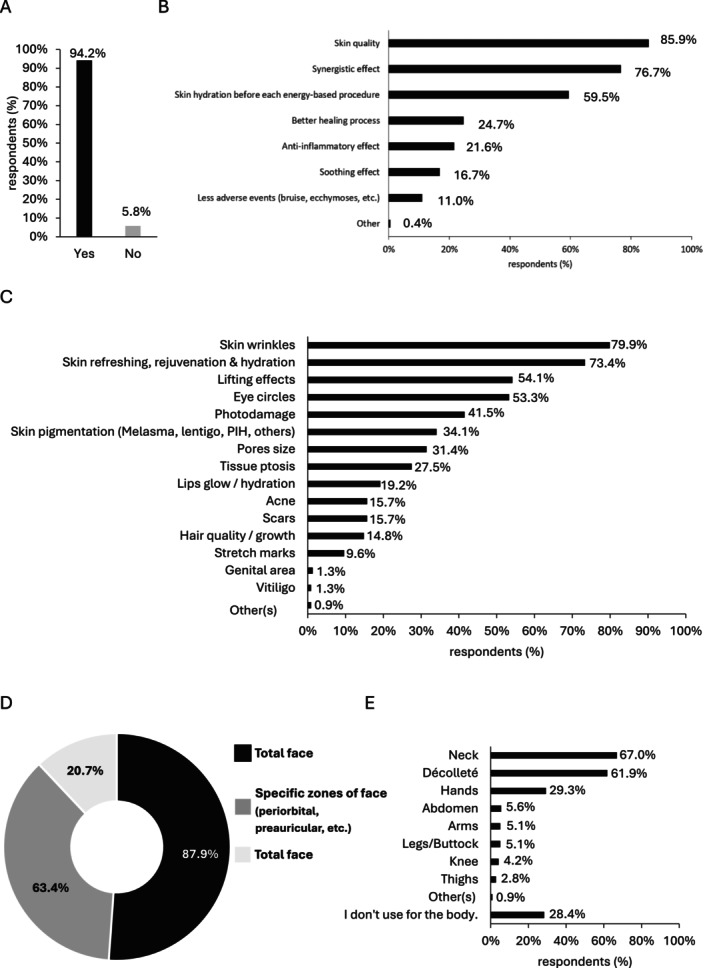
NCTF135HA used in combination for aesthetic procedures. (A) Percentage of respondents who are combing NCTF135HA for aesthetic procedures (*n* = 296; single‐choice question); (B) Reasons to combine NCTF135HA for aesthetic procedures (*n* = 227; multiple‐choice question), Other: “Improving the response of the bio‐scaffold by creating a better extracellular matrix environment”; (C) Indications for aesthetic procedures in which NCTF135HA is combined (*n* = 229; multiple‐choice question), Other(s): Epidermal cell remodeling; (D) Areas treated with NCTF135HA in combination therapy (*n* = 232; multiple‐choice question); (E) Body areas treated with NCTF135HA in combination therapy (*n* = 215; multiple‐choice question), Other(s): Lips and vuvla.

The majority of the respondents (94.2%, 276/296) utilized NCTF135HA in combination therapy for aesthetic purposes (Figure 3A). Among these practitioners, 85.9% (195/227) combined NCTF135HA to enhance skin quality and 76.7% (174/227) to achieve a synergistic effect (Figure 3B). The primary indications for combining NCTF135HA with other aesthetic procedures were skin wrinkles (79.9%, 183/229) and skin refreshing (76.7%, 168/229) (Figure 3C). Respondents predominantly (87.9%, 204/232) combined NCTF135HA with other aesthetic procedures for the entire face (Figure 3D), although 63.4% (147/232) also used it for specific areas like the periorbital and periauricular regions, and 20.7% (48/232) applied it for body treatments (Figure 3D). Among those using NCTF135HA for body treatments, 67.0% (144/215) focused on the neck, and 61.9% (133/215) targeted the décolleté area (Figure 3E).

**FIGURE 4:**
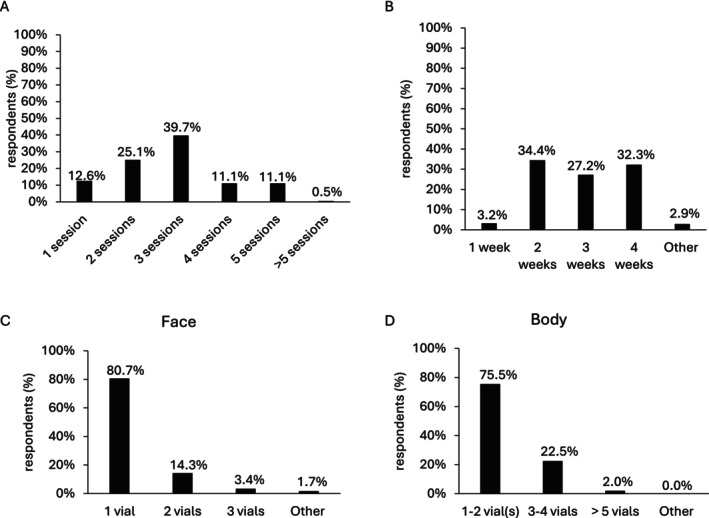
NCTF135HA frequency and volume. (A) Number of sessions to inject NCTF135HA in combination therapy for aesthetic purposes (*n* = 199; multiple‐choice response); (B) Frequency of the sessions (*n* = 278; multiple‐choice question), Other: Up to 6 weeks, up to 8 weeks, up to 3 months, Day 0, 15, 15, 1 month later, 1 month later, the first two sessions every 2 weeks, and the following 3 sessions every 4 weeks; (C) Number of NCTF135HA vials used for the face (*n* = 238; multiple‐choice question), Other: 1 to 2 mL, it depends on the protocol; (D) Number of NCTF135HA vials used for the body (*n* = 152; multiple‐choice question).

Approximately 40% (79/199) of practitioners reported administering NCTF135HA over three sessions, while 12.6% (25/199) and 25.1% (50/199) opted for one and two sessions, respectively (Figure 4A). Moreover, 11.1% (22/199) of respondents utilized NCTF135HA for combination therapy across four or five sessions (Figure 4A). All of the practitioners space NCTF135HA injection session between 2 to 4 weeks (every 2 weeks by 34.4% (96/279) of practitioners, every 3 weeks by 27.2% (76/279), and every 4 weeks by 32.3% (90/279)) (Figure 4B). For facial treatments, a majority (80.1%, 192/238) utilized a single vial of NCTF135HA, while for body treatments, practitioners typically employed one to two vials (75.5%, 114/151) (Figure 4D).

##### 3.3.2.1 Combination With Injectable Products

**FIGURE 5:**
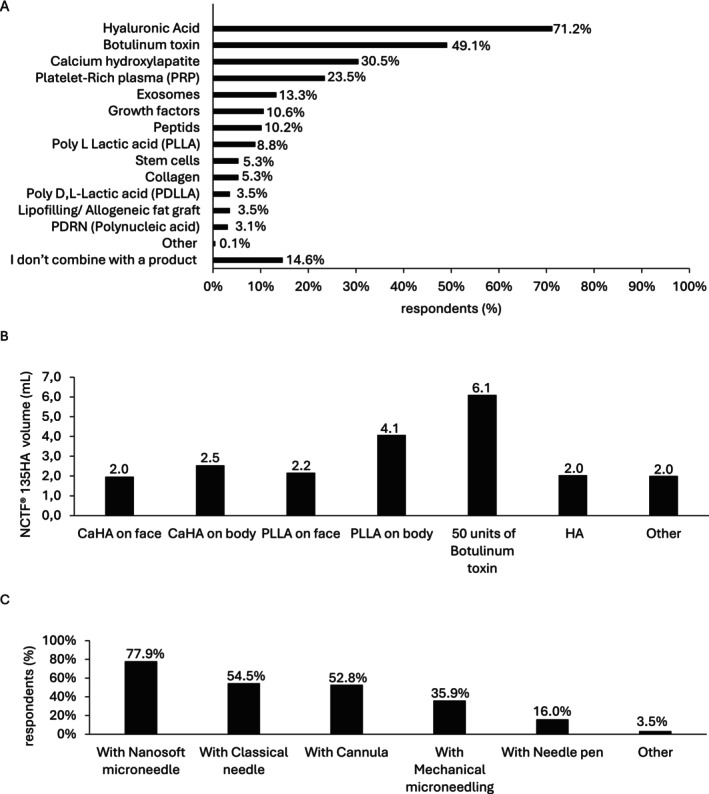
NCTF135HA combination with injectable products. (A) Type of injectable products used in combination therapy for aesthetic purposes (=226; multiple‐choice question), Other: Smooth polydioxane threads; (B) Volume of NCTF135HA used with different injectable products (*n* = 118; open‐field response), Other: 1 to 3 mL with PRP, 1 mL for 20 mL of fat, 1 mL with peptides, 2 mL with Aquagold, 2 mL with polynucleotides; (C) Type of instruments used to inject (*n* = 231; multiple‐choice question), Other: Mesotherapy needles.

When combined with injectable products, 71.2% (161/226) and 49.1% (111/226) of respondents reported combining NCTF135HA with HA and botulinum toxin, respectively (Figure 5A). On average, practitioners combined 2.0 mL (median: 1.75 mL; min‐max [0–10]) of NCTF135HA with HA (Figure 5B). When combined with 50 units of botulinum toxin, practitioners typically used an average of 6.1 mL (median: 3 mL; min‐max [0–100]) of NCTF135HA (Figure 5B). For calcium hydroxylapatite (CaHA), practitioners used an average of 2.0 mL (median: 1.5 mL; min‐max [0–6]) of NCTF135HA for facial treatments and 2.5 mL (median: 2 mL; min‐max [0–6]) for body treatments (Figure 5B). When combining with poly‐L‐lactic acid (PLLA), practitioners used an average of 2.2 mL (median: 1.5 mL; min‐max [0–20]) of NCTF135HA for the face and 4.1 mL (median: 2 mL; min‐max [0–20]) for the body (Figure 5B). Additionally, the majority of respondents injected NCTF135HA using nanosoft microneedles (77.9%, 180/231), classical needles (54.5%, 126/231), and cannulas (52.8%, 122/231) (Figure 5C).

##### 3.3.2.2 Combination With Technologies

**FIGURE 6:**
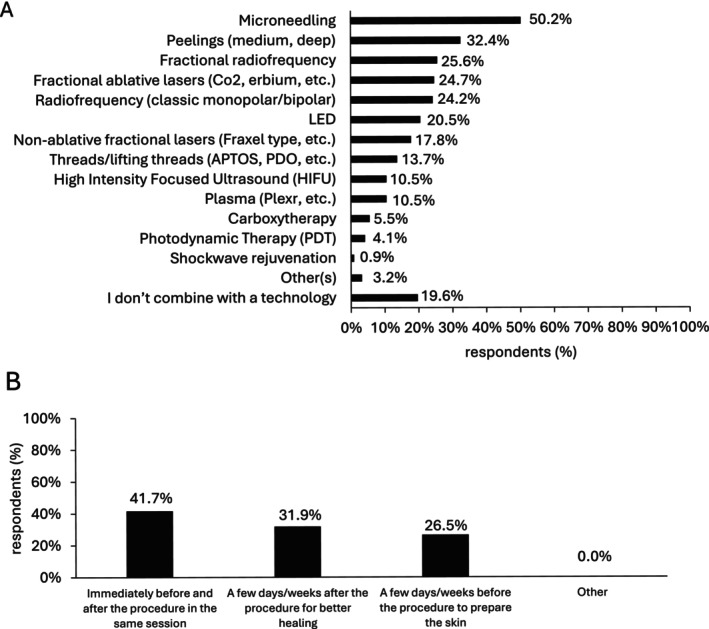
NCTF135HA in combination therapy with technologies. (A) Technologies used in NCTF135HA combination therapy (*n* = 219; multiple‐choice question), Other(s): Microfocused ultrasound, laser technology (Diode endolaser, K laser, Intense pulse light), Aquagold and Jett plasma; (B) NCTF135HA injection protocol for combination with energy‐based devices (*n* = 204; multiple‐choice response).

In combination with technologies, NCTF135HA was utilized by 50.2% (110/219) of respondents with microneedling technology, 32.4% (71/219) with peelings, 25.6% (56/219) with Fractional Radiofrequency, 24.7% (54/219) with fractional ablative lasers, and 24.2% (53/219) with classic Radiofrequency (Figure 6A). Additionally, almost 20% (43/219) of practitioners specified not combining NCTF135HA with technologies, but rather with a product and/or surgery (Figure 6A). Among those integrating NCTF135HA with energy‐based devices, 41.7% (84/204) initiated treatment immediately before and after the procedure within the same session (Figure 6B). Moreover, 31.9% (65/204) chose to start treatment a few days/weeks after the procedure to enhance healing, while 26.5% (54/204) opted to begin treatment a few days/weeks before the procedure to prime the skin (Figure 6B).

##### 3.3.2.3 Combination With Surgery

**FIGURE 7:**
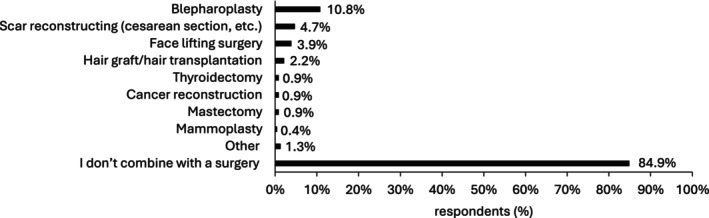
NCTF135HA combination with surgery. (Multiple‐choice question; Other: Labioplasty, vulvar harmonization, Brazilian Butt Lift/Fat Graft).

9.7% of the respondents were surgeons (Table 1). A large majority of respondents (84.9%, 197/232) declared not combining NCTF135HA with surgery (Figure 7). 10.8% (25/232) were combining NCTF135HA with blepharoplasty, 4.7% (11/232) with scar reconstruction and 3.9% (9/232) with face lifting surgery (Figure 7).

## 4 Discussion

### 4.1 Findings and Interpretations

NCTF 135HA is used since 1978 and its selling rate is about 10 vials per minute according to the company data. This international practice survey aimed to investigate the real‐life application of this product alone or in combination therapy worldwide, providing insights into its use in both aesthetic and clinical contexts.

Polyrevitalization combinations are primarily used for rejuvenation, wrinkle treatment, and addressing photodamaged skin [5, 9, 15, 21, 22, 23, 24]. In agreement with this observation, NCTF135HA used alone is indicated to treat fine lines, hydration, radiance, and elasticity [31]. Thus, it is not surprising that the majority of healthcare providers using NCTF135HA although in combination therapy, are still following these indications.

Following the 2023 publication by Theodorocopoulou et.al., practitioners gained a clearer understanding of how a polynutrient solution can create an effective scaffold in the extracellular matrix. This advancement enables better stimulation of skin cells, especially fibroblasts, leading to improved production of elastic fibers and collagen [23]. This scaffold is necessary for almost all biostimulators such as Energy‐based Devices, Threads, liquid biostimulators. In addition, data confirm that integrating multiple aesthetic therapies, each targeting different aspects of the aging process, yields optimal results. This approach not only enhances overall efficacy but also leads to higher levels of patient satisfaction [32]. Thus, according to the survey respondents, the primary reasons for using NCTF135HA in combination aesthetically are to improve skin quality, achieve a synergistic effect with other treatments, and hydrate the skin prior to energy‐based procedures. Consistent with this, there is increasing evidence indicating that combining NCTF135HA with injectable products such as botulinum toxin and HA, or with technologies like nanochips, notably enhances skin quality and hydration [6, 7, 26, 27]. Furthermore, its safety was validated in a prospective, multicenter study involving 99 patients who received NCTF135HA in combination with ART FILLER Fine Lines and/or ART FILLER Universal [7]. The study, which was nonrandomized and noncomparative, reported no serious adverse events and 67 adverse events were reported, among them, 57 were expected and commonly associated with dermal injections. Nonetheless, some respondents stated not combining NCTF135HA because of limited experience, cost concerns, or uncertainty regarding potential synergistic effects. This underscores the importance of providing comprehensive information and training to physicians worldwide on this topic.

HA and botulinum toxin are widely employed for skin tightening, face rejuvenation, and wrinkle reduction, making them among the most frequent products for aesthetic medicine [6, 10, 33]. Previous studies confirmed the interest of combination of polyrevitalizing products with botulinum toxin [34]. Combining either HA or botulinum toxin with NCTF135HA likely enhances their effects on skin rejuvenation and wrinkle filling [6, 7]. The frequent combination of NCTF135HA with HA (71.2%) and botulinum toxin (49.1%) among survey respondents highlights the widespread adoption of these therapies in clinical practice, indicating consensus on their effectiveness.

In clinical practice, combination therapy is widely adopted for the treatment of conditions such as melasma and rosacea [35, 36, 37]. Notably, a commonly utilized combination for melasma treatment includes hydroquinone, topical steroids, and retinoic acid [35]. The composition of NCTF135HA, comprising vitamins, nucleosides, amino acids, minerals, co‐enzymes, antioxidants, and HA [31], underscores its potential efficacy in addressing dermatological conditions. The fact that more than half of practitioners employ NCTF135HA in combination therapy for medical purposes highlights its value as a beneficial tool in managing conditions like melasma and rosacea. These findings collectively suggest that NCTF135HA could be promising as a component in the treatment of dermatological conditions, extending beyond the aesthetic considerations.

In the present study, practitioners declared favoring microneedling (50.2%), peelings (32.4%), fractional radiofrequency (25.6%), and lasers (24.7%) when combining NCTF135HA with technologies. Although no clinical trials have directly addressed the efficacity of NCTF135HA combination with technologies, many studies have supported the benefits of technology combination [38, 39]. These advanced techniques provide less invasive solutions for skin rejuvenation, scar management, wrinkle reduction, and the improvement of skin texture and tone [40, 41, 42, 43, 44, 45, 46, 47, 48, 49]. Additionally, combination of such technologies together or with topical agents and surgery has been shown to increase their efficiency without altering safety of the procedures. For instance, microneedling combination with HA, has been shown to improve the penetration and efficacy of topical agents like HA [50]. In regard to NCTF135HA, its combination with nanochips microneedling improves skin texture and brightness [26].

According to the recommendations, the optimal protocol for NCTF135HA injection involves three sessions spaced 2 weeks apart [31]. However, despite the majority adhering to this protocol (39.7%), survey responses demonstrated a heterogeneity of users, with 12.6% and 25.1% opting for one or two sessions, respectively. Additionally, NCTF135HA injections are administered at varying intervals: 34.4% of practitioners administer them every 2 weeks, 27.2% every 3 weeks, and 32.3% every 4 weeks, highlighting significant diversity in clinical practice. However, this variation is totally acceptable because of the clinical trial proved the interval of 21 days ±3 days which is conformed to 2 to 4 weeks [21]. Among potential factors contributing to this heterogeneity, variations in patient and doctors' availability for multiple treatment sessions likely play significant roles. Furthermore, a literature review examining combination treatments involving RF, intense pulse light, non‐ablative and ablative lasers, as well as fillers or botulinum toxin, demonstrated the safety and effectiveness of same‐day combined procedures for rejuvenation [51]. These combined treatments not only enhance clinical outcomes but also provide greater comfort for patients without any apparent loss of efficacy or adverse effects. Although the review did not include NCTF135HA among the combination treatments investigated, it can be argued that similar efficacy and safety could be demonstrated. Published data have confirmed its effects on different skin cells as a cell medium [49, 52]. Continued research into the safety and efficacy of same‐day combined procedures for rejuvenation is essential for standardizing healthcare providers' practices.

Approximately 85% of respondents opt against utilizing NCTF135HA alongside surgical procedures. This trend can be elucidated by the predominant presence of aesthetic doctors (52.1%) among the respondents, who typically do not perform surgeries, whereas surgeons comprise only 9.7%. Moreover, the absence of clinical trials substantiating the advantages of combining NCTF135HA therapy with surgical interventions could argue for this low frequency of application.

Importantly, our survey analysis did not reveal significant variations in combination practices among respondents based on factors such as country, specialty, or level of experience (sub‐analyses not shown), suggesting a consensus in the use of NCTF135HA in combination therapy across diverse demographics. This uniformity underlines the widespread acceptance and effectiveness of NCTF135HA combination as a versatile component in various aesthetic and clinical treatments.

### 4.2 Strengths and Weaknesses

The survey was distributed to professional networks by Scientific Committee members and local learned societies. As a result, the target group was well represented, drawing participants from around the globe. However, one limitation was the large number of respondents from Mexico, comprising 30.8% (*n* = 158/507) of the total respondents. In addition, most of the respondents stated having less than 10 years of experience. Nonetheless, we observed a uniformity in survey responses when comparing answers based on nationality or experience. Although NCTF135HA is not approved in the US or Canada, three respondents (2 from Canada, 1 from US) reported using the polyrevitalizing solution in classic biorevitalization and combination therapy, indicating off‐label use.

### 4.3 Similarities and Differences in Relation to Other Studies

To our knowledge, this is the first survey about NCTF135HA combination therapy international practice worldwide conducted among healthcare professionals.

## 5 Conclusion

The combination therapy is a proved approach in aesthetic medicine. Nowadays, practitioners prefer to improve clinical efficacy of routine aesthetic procedures with a polyrevitalizing solution which provides a scaffold of micronutrients to stimulate skin cells particularly fibroblasts. NCTF 135HA has a relevant scientific background which encourage the practitioners to combine it with other procedures. There is a lack of performance and safety data for each combination mentioned in this survey in order to be able to standardize this type of approach.

## Author Contributions

F.F., G.C., E.B., H.C., M.L., H.G., F.B., A.O., E.T., A.A., H.G., P.H.P., I.G.L., G.R., N.S., H.I., V.P., A.S. reviewed the literature and drafted the questionnaire and of the publication. F.F., N.S., H.I., V.P., A.S., M.C., S.L.C., and A.A.V. reviewed the literature, analyzed and synthesized the questionnaire results, and drafted of the publication.

## Conflicts of Interest

F.F., N.S., H.I., and V.P. are the employee of Laboratoires FILLMED. The other authors have no conflict of interest to declare.

## Data Availability

The data that support the findings of this study are available on request from the corresponding author. The data are not publicly available due to privacy or ethical restrictions.
